# An Example of a Stroke Unit Reshaping in the Context of a Regional Hub and Spoke System in the COVID-19 Era

**DOI:** 10.3389/fneur.2020.01029

**Published:** 2020-10-15

**Authors:** Elisa Candeloro, Federico Carimati, Payam Tabaee Damavandi, Lucia Princiotta Cariddi, Paola Banfi, Alessandro Clemenzi, Marco Gallazzi, Marco Mauri, Valentina Rebecchi, Fabio Baruzzi, Andrea Giorgianni, Matteo Tozzi, Massimo Bianchi, Walter Ageno, Maurizio Versino

**Affiliations:** ^1^Neurology and Stroke Units, ASST-Settelaghi, Ospedale di Circolo Varese, Varese, Italy; ^2^Department of Medicine and Surgery, Bicocca University, Milan, Italy; ^3^Department of Medicine and Surgery, Insubria University, Varese, Italy; ^4^Neuroradiology Unit, ASST-Settelaghi, Ospedale di Circolo Varese, Varese, Italy; ^5^Vascular Surgery Unit, ASST-Settelaghi, Ospedale di Circolo Varese, Varese, Italy; ^6^Emergency Department, ASST-Settelaghi, Ospedale di Circolo Varese, Varese, Italy

**Keywords:** stroke unit, reshaping, hub and spoke system, COVID-19 pandemic, timing

## Abstract

During the COVID-19 outbreak, the Neurology and Stroke Unit (SU) of the hospital of Varese had to serve as a cerebrovascular hub, meaning that the referral area for the unit doubled. The number of beds in the SU was increased from 4 to 8. We took advantage of the temporary suspension of the out-patient clinic and reshaped our activity to guarantee the 24/7 availability of recombinant tissue Plasminogen Activator (rtPA) intravenous therapy (IVT) in the SU, and to ensure we were able to admit patients to the SU as soon as they completed endovascular treatment (EVT). In 42 days, 46 stroke patients were admitted to our hospital, and 34.7% of them underwent IVT and/or EVT, which means that we treated 0.38 patients per day; in the baseline period from 2016 to 2018, these same figures had been 23.5% and 0.23, respectively. The mean values of the door-to-first CT/MRI and the door-to-groin puncture, but not of the onset-to-door and the door-to-needle periods were slightly but significantly longer than those observed in the baseline period in 276 patients. On an individual basis, only one patient exceeded the door-to-groin puncture time limit computed from the baseline period by about 10 min. None of the patients had a major complication following the procedures. None of the patients was or became SARS-CoV2 positive. In conclusion, we were able to manage the new hub-and-spoke system safely and without significant delays. The reshaping of the SU was made possible by the significant reduction of out-patient activity. The consequences of this reduction are still unknown but eventually, this emergency will suggest ways to reconsider the management and the allocation of health system resources.

## Introduction

Since the outbreak of COVID-19 in Lombardy, an Italian region with a population of 10 million people. During the pandemic, the ability to guarantee treatment for patients presenting with stroke within the time windows dictated by guidelines has become an issue. This is because most of the resources normally available in hospitals had to be devoted to the treatment of COVID-19 patients.

To face the problem of cerebrovascular and other time sensitive diseases, the Governor of Lombardy set up a hub-and-spoke organization with 10 hub hospitals. It is noteworthy that this decision was taken in a few days, with little time for the hub hospitals to reorganize their activity. The hospital of Varese had to serve as a cerebrovascular hub for the north-western areas of Lombardy, meaning that its referral area was doubled and that the stroke patients could have been taken to Varese either directly by the regional emergency transportation system (Agenzia Regionale dell'Emergenza Urgenza: AREU) or from a spoke hospital located somewhere in Lombardy.

Here we report how we have managed this situation and how our Stroke Unit (SU) was able to maintain consistent performance levels whilst also becoming a hub.

## Materials and Methods

### Reshaping

Before the onset of the COVID-19 pandemic, the Neurology and Stroke Unit of the Circolo Hospital in Varese consisted of 14 beds. Four beds were dedicated to the Stroke Unit since there were four mobile monitoring systems available. The monitors were placed next to the patient's bed and could not be remotely controlled. The medical staff consisted of 8 full-time neurologists who belonged to the hospital and one half-time neurologist who belonged to the University of Insubria. With different levels of involvement, all neurologists worked in the Stroke Unit but none of them was exclusively assigned to it. During the week, from 8 a.m. to 8 p.m. (daytime) at least one neurologist had to be present in the hospital, whereas from 8 p.m. to 8 a.m. (night-time) one neurologist was available on call. On the weekends there was a neurologist in the hospital during daytime and one on-call during night-time. The nurse staff was shared with another 3 units located on the same floor. In total, 11 nurses dealt mainly with the Neurology and Stroke Unit, 8 of which were specifically trained for the Stroke Unit. Intravenous Therapy (IVT) with recombinant tissue Plasminogen Activator (rTPA) procedures were performed in the Emergency Department (ED) whilst Endovascular Therapy (EVT) procedures were conducted in the angiographic room. After the procedures the patients were moved to the Stroke Unit, kept under observation in the ED, and if a major complication occurred or the patient was clinically unstable, they were moved to the Intensive Care Unit (ICU).

Immediately after the promulgation of the decree, the Neurology and SU were relocated in the nearby cardiac surgery ward, since most of the cardiac surgeons had to move to another hospital that served as a hub for cardiac surgery. Thanks to this relocation, the SU gained 4 additional beds that were provided with a centrally and remotely controlled monitoring system. The number of neurologists was not increased but, given the substantial reduction of the outpatient clinic activity that was imposed by the lockdown, it was possible to reorganize the neurology department as follows: during the week, three neurologists were present in the SU during day-time, two of which were present in the ward, and one in the ED. In addition, one neurology resident was available 3 out of 5 weekdays. During night-time, one neurologist was on call and another one was available as a possible back-up. On the weekends, during the daytime, there was one neurologist and one resident available both for the Neurology and Stroke Unit and for the ED. During night-time, one neurologist was available on-call with an additional neurologist as a possible backup. As for the nurses, eight nurses were available during day-time and two at night-time.

The regional indications dictated the rules for swab testing and for the personnel protection equipment that are described in a report on our hospital's neurosurgery hub (1). The swab test became mandatory for all the patients admitted to the hospital only after April 14. Before that date, a swab test was performed on patients that were possibly considered to be SARS-CoV2 positive based on their clinical history, their body temperature, their respiratory symptoms and signs, and a chest x-ray (or CT).

The IVT procedures were performed inside the Stroke Unit and not in the ED. All patients admitted to the ED were transferred to the SU immediately after they underwent IVT or EVT or when neither of these procedures was deemed possible or appropriate unless they had to be transferred to the ICU. This was done to alleviate the burden on the ED.

### Data Collection and Analysis

We collected the data on the patients that were referred to our hospital from March 9 to April 19 2020 either for ischemic or hemorrhagic stroke or for intracerebral cerebral hemorrhage (ICH), i.e., a timeframe of 42 days following the promulgation of the decree of the Lombardy Governor for the institution of the hub-and-spoke system.

Since the data were collected from patients' clinical records, and since they were all treated according to guidelines on best clinical practice, our institution did not require ethical approval for this study.

We performed a full diagnostic work-up on all patients and when indicated by Italian guidelines [Spread GL 2017 (2)], updated with the most recent AHA/ASA guidelines (3), an IVT and/or an EVT or a carotid endarterectomy (CEA).

For each patient we acknowledged how he/she had reached the hospital (without or with the regional emergency transportation system AREU), the individual risk factors, the kind of stroke according to the Trial of Org 10172 in Acute Stroke Treatment (TOAST) classification (4), the location of the stroke according to Oxfordshire Classification (OCSP) (5), the therapeutic procedures (IVT, EVT, CEA), and a justification in case no procedure was undertaken, the NIH Stroke Scale (NIHSS) score before and after the procedure. We also acknowledged several time periods: onset-to-door (the time from the onset of the symptoms to the arrival at the ED), door-to-first CT/MRI (the timeframe from the arrival of the patient at the hospital and the first neuroradiological procedure), door-to-needle (the timeframe from the arrival of the patient at the hospital and the beginning of IVT), and door-to-groin puncture (the timeframe from the arrival of the patient at the hospital and the beginning of EVT).

We defined as a baseline the data that has been collected from 276 patients over a 3 year period, from 2016 to 2018, and for the different time periods we defined the 95th percentile value as the upper normal (i.e., not COVID-19) limit.

For the evaluation of the mean values computed for the COVID-19 period, we calculated a z-value by considering the mean and the standard deviation values computed for the baseline period as the population values, and the number of observation in the COVID-19 period as the numerosity value.

For the comparison of observed frequencies computed for the COVID-19 period, we computed chi-square values using the corresponding frequencies during the baseline to compute the expected frequencies.

For the comparison of the variabilities, we computed an F value as the ratio between the baseline and the COVID-19 variances.

All the analyses were performed using Microsoft Excel ver. 16.39, and for all of them the significance value was set at *p* = 0.050.

## Results

We did not turn down any case requests for the admission of a patient referred to our hospital.

In the 42 days following the promulgation of the decree, we observed 52 patients: 6 TIAs and 46 strokes. In the same timeframe, 35 ICH patients were admitted to the hospital, mostly in the intensive care and in the neurosurgery units, while only 2 in the Neurology and Stroke Units.

Sixteen of these 46 patients (34.7%) underwent a revascularization procedure: 3 patients (18.8%) had IVT, 8 (50%) had EVT, and 5 patients (31.2%) had IVT followed by EVT (Bridging treatment). Of the patients treated during the COVID-19 period, 34.7% were found to be significantly higher when compared to the baseline period, where only 23.5% of patients were treated (chi-square = 4.4; *p* = 0.037).

We thus treated 0.38 stroke patients per day, whereas the corresponding figure for the baseline period was 0.23; again, these two figures proved to be significantly different (chi-square = 5.40; *p* = 0.002).

None of these patients had a major complication following the revascularization procedure, but 2 patients had to be admitted to the ICU for a few days before being transferred to the SU. No patients were or became SARS-CoV2 positive. All of these patients reached the hospital by ambulance after the activation of the AREU system.

The main clinical features of the patients who underwent to recanalization procedures are reported in Table 1.

**Table 1:** Main clinical features of patients who underwent IVT and EVT recanalization procedures.

Gender (*n*; %)	Male	8	50%
	Female	8	50%
Age (median; range)		77	42–92
OCSP (*n*; %)	Partial Anterior Circulation Infarct	5	31.2%
	Total Anterior Circulation Infarct	6	37.5%
	POsterior Circulation Infarct	4	25%
	LACunar Infarct	1	6.3%
TOAST (*n*; %)	Large vessel	2	12.5%
	Cardioembolism	9	56.2%
	Small vessel	1	6.2%
	Other or undetermined	4	25%
mRS (median; range)	Pre-stroke	0	0–1
	At discharge	2	0–5
NIHSS (median; range)	Onset	10.5	2–25
	After 24h	4.5	1–25
	At discharge	2	0–5

*mRS, modified Rankin Score*.

The following stroke risk factors were found in the patients treated: atrial fibrillation in 8 (50%), ischemic heart disease in 5 (31.3%), diabetes in 3 (18.8%), a smoking habit in 6 (37.5%), hypertension in 12 (75%), previous stroke in 3 (18.8%), time-based clinical history of a previous TIA in 2 (12.5%).

Table 2 shows the interventional time periods. The mean values of door-to-CT/MRI and door-to—groin puncture were slightly, but significantly, longer than those measured in the large case series of 276 patients collected from 2016 to 2018, whereas the mean values of onset-to-door and door-to-needle were not. However, it is worth mentioning that if we considered the normal upper time limit computed from the data of the baseline period, only one patient exceeded the door-to-groin puncture time limit by about 10 min.

**Table 2:** The table reports the mean and the standard deviation (STD) of the different interventional time periods measured in the COVID-19 and the Baseline (from 2016 to 2018) periods.

	**COVID-19**		**Baseline (2016–2018)**					
**Time periods (min)**	**Mean**	**STD**	**Mean**	**STD**	***z***	***p***	**Baseline upper limit**	***N* (%) of patients exceeding upper reference limit**
Onset–to-door	84.1	21.4	86.2	46.44	0.18	0.850	178	0 (0%)
Door-to-first CT/MRI	54.9	18.5	30.62	31.62	3.07	0.002	108	0 (0%)
Door-to-needle	100.7	40.4	77.39	36.12	1.81	0.070	167.7	0(0%)
Door-to-groin puncture	189.1	49.2	137.12	75.13	2.49	0.010	308.8	1 (6.2%)

*The z and p-values refer to the comparisons of the means*.

Another interesting point is that some of the data presented a larger variability in the baseline period compared to the COVID-19 period, as showed by the variances of the mean. This was true for the onset-to-door (*F* = 4.7; *p* < 0.001), the door-to-first CT/MRI (*F* = 2.92; *p* < 0.001), the door-to-groin puncture (*F* = 2.33; *p* = 0.005) but not for the door-to-needle (*F* = 0.9; *p* = 0.600) timeframes.

Four additional patients underwent CEA. They were 3 males and 1 female and their median age was 71 years with a range of 55–88 years. They all had a partial anterior circulation infarct and their median NIHSS score was 1 at onset (range 0–2) and 0 at discharge (0–1). Their mean (and standard deviation) values for the onset-to-door and the door-to-first CT/MRI were, respectively 109 (80) and 78 (53) min. We exceeded the normal time limit in one patient for the onset-to-door and in another patient for the door-to-CT/MRI time period. The time between the admission and the surgical procedure ranged from 2 to 10 days.

Among the patients who did not undergo any revascularization procedure, eight (27%) could not be treated due to inappropriate timing [i.e., onset-to-door larger than 6 h without DAWN/DEFUSE-3 trials criteria (6–8)]; in the baseline period, the corresponding figure was the same (26%). Four of these patients did not activate the emergency system to get to the hospital.

## Discussion

The outbreak of COVID-19 has had a significant impact on health systems worldwide. The involvement of the nervous system in the SARS-CoV2 virus is now recognized (9, 10), and endothelial involvement is likely to play a key role (11–15). Thus, the management of stroke in the COVID-19 era has two aspects: one concerning COVID-19-related stroke (16, 17), and another about the need to meet the standards for the treatment of a time-dependent disease, despite having to allocate health system resources to the management of COVID-19. The latter had to be faced locally, and the reports about this topic were not available at the time of the pandemic onset (17–19).

Our data showed that despite the fact that the COVID-19 pandemic imposed a reallocation of health system resources, largely toward the management of COVID-19 patients, we were still able to guarantee a timely and safe approach for stroke patients. Eventually, we had no COVID-19 patients, but we still adopted an approach to safeguard them from potentially SARS-CoV2 positive patients.

Due to the hub-and-spoke system, the number of patients that underwent a recanalization procedure, and the percentage of patients eligible for treatment increased significantly. The onset-to-door and the door-to-needle mean time periods did not change as compared to the baseline period, whereas the door-to first CT/MRI and the door-to-groin puncture were slightly but significantly increased. On an individual basis, only one patient exceeded the door-to-groin puncture limit by about 10 min. The increase of the mean values was expected due to the additional safeguard measures that had to be adopted for potentially SARS-CoV2 positive patients. However, the variability of the duration of the different time periods was usually shorter in the COVID-19 than in the baseline period, suggesting that the control of the sequence of the procedures was improved.

This was made possible by the reshaping of the SU in terms of both equipment and human resources. The hospital increased the number of monitored beds available in the SU. The neurologists could focus on inpatient activity since the activity of the outpatient clinic was reduced and limited to those presenting for an acute or subacute problem. Moreover, during the pandemic, patients were reluctant to be referred to the hospital because they were afraid of being infected, as suggested by the unexpected reduction of consultations for cardiovascular disorders.

It is possible that when we go back to regular activity, we will find that many patients have underestimated their neurological symptoms. This could be the case of TIA, which can last a short time and be overlooked. The number of patients admitted for a TIA was quite low, in agreement with the report by Diegoli et al. (20). However, in the 3 months after the time period considered in this report, none of the patients that we admitted for a stroke had a clinical history positive for TIA. If in the future we confirm this scenario, we should reconsider how medical resources are distributed. In Italy we there is an issue of overuse of medical resources, and it is not unusual for a patient to have several medical consultations, laboratory, and instrumental examinations before concluding that “there is nothing wrong.” Often, this conclusion could have been reached with a thorough initial examination and less referrals.

In conclusion, it is possible that this emergency period, which forced us to activate different procedures, will provide suggestions that will enable us to reconsider organization and lead to the implementation of more hub-and-spoke systems, to the reweighting of the out- and in-patient activities, and, therefore, to more careful examinations of the patient before asking for further clinical and instrumental examinations.

## Data Availability Statement

The raw data supporting the conclusions of this article will be made available by the authors, without undue reservation.

## Ethics Statement

Ethical review and approval was not required for the study on human participants in accordance with the local legislation and institutional requirements. Written informed consent for participation was not required for this study in accordance with the national legislation and the institutional requirements.

## Author Contributions

All authors contributed to the management of the patients, conceived the study, contributed to the draft manuscript, and read and approved the final version. Moreover, PT and FC collected the data in a suitable database for the following analyses. MV analyzed the data and wrote the manuscript.

## Conflict of Interest

The authors declare that the research was conducted in the absence of any commercial or financial relationships that could be construed as a potential conflict of interest.
